# The beautiful game bringing families together: children’s and parents’ experiences of a family football programme

**DOI:** 10.1093/heapro/daad183

**Published:** 2024-01-09

**Authors:** Melissa A Fothergill, Gursharan Gill, Pamela L Graham

**Affiliations:** Department of Psychology, Northumbria University, Northumberland Road, Newcastle-upon-Tyne, NE1 8ST, UK; School of Psychology,Newcastle University, Wallace Street, NE2 4DR, UK; Department of Social Work, Education and Community Wellbeing, Northumbria University, Coach Lane Campus, NE7 7XA, UK

**Keywords:** physical activity, nutrition, children, parents, football

## Abstract

Children are consistently not achieving recommended levels of physical activity (PA) despite it being a compulsory requirement of the national curriculum in England. Fruit and vegetable consumption also falls below recommended levels for both adults and children. With school PA increasingly being outsourced, football foundations (linked to professional football clubs) are now prominent providers of children’s PA. However, research exploring coach-led interventions in schools is limited with a particular gap in knowledge surrounding the qualitative experiences of children and parents/carers. The current study therefore aimed to explore the experiences of children and parents engaged in a 6-week family football programme. Family football is a free after-school programme engaging parents/carers and their children, to enhance their engagement in PA and healthy nutritional habits. A purposive sample of parents/carers and their children (*N* = 36) took part in qualitative focus groups to discuss their experiences of participating in family football. Reflexive thematic analysis revealed two themes: (i) healthy lifestyle facilitators and challenges and (ii) added value of programme participation. Children discussed programme content around nutritional practices and parents/carers discussed examples of where nutritional practices had improved at home, though some parents/carers and children highlighted persistent barriers to health improvement. Both parents/carers and children reflected on how the programme had enabled them to spend dedicated time with one another. The findings emphasize the significance of direct parent/carer involvement in school-based health interventions as well as the value of coach-led interventions in the enhancement of PA enjoyment, nutritional knowledge and connectedness of families within schools.

**Contribution to Health Promotion**

- Provides insight into parents’ and children’s experiences of a real-world physical activity and nutrition programme.
- Shows how sporting organizations can be used by primary schools to engage children and parents in health education.
- School-based activities delivered to families using the medium of sport can enhance short-term parent–child relationships.

## INTRODUCTION

It is widely documented that children should participate in 60 min of moderate-to-vigorous physical activity per day (MVPA) (Chief Medical Officer, 2019). Recent systematic reviews have shown that engagement in physical activity (PA) can be beneficial to children’s physical and mental health, and cognitive performance (Bidzan-Bluma and Lipowska, 2018; Rodriguez-Ayllon *et al.*, 2019; Smith *et al.*, 2019). Given the well-established benefits of PA, physical education (PE) is compulsory in schools in England across the national curriculum at all stages (Long and Roberts, 2019). Despite this compulsory requirement, survey data from England reported that 53.2% of 5- to 16-year-old children were not achieving recommended PA levels (Sport England, 2019). Subsequently, low levels of PA in children and young people have been associated with poor mental and physical well-being as well as poorer school performance (Janssen and Leblanc, 2010; Bailey *et al.*, 2013). In the UK, primary school teachers are typically generalist and consequently are not experienced or trained in the delivery of PA (Sloan, 2010; Domville *et al.*, 2018). Therefore, in 2013–2014 ring-fenced funding from the Soft Drinks Industry Levy was provided by the UK Government’s Primary PE and Sport Premium until 2021–2022 for schools to fund facilities, healthy eating and after-school clubs (Long and Roberts, 2019). Due to the lack of primary school teachers having a subject specialism in PE, this funding has resulted in an upsurge in the employment of coaches and external companies to fully deliver or partially contribute to PA provision (Jones and Green, 2017). Insightful research which examined the role of specialist coaches working alongside generalist teachers in the provision of PE in England, reported that the involvement of supportive and skilled coaches can positively impact children’s level of enjoyment and engagement in PE. While children liked that their teachers were involved in their lessons, they commented that teachers could be passive and struggle to tailor activities to their abilities in comparison to coaches. Indeed, primary school educators perceived school-based PA as important for children’s development, but despite these benefits, they identified lack of time, poor facilities and pressure as barriers (Domville *et al.*, 2018, 2019). Moreover, it has been argued that schools inadvertently promote sedentary behaviour by focusing heavily on activities that require children to remain seated for much of the day (Weiler *et al.*, 2014). Notwithstanding, schools are still often viewed by a range of stakeholders as viable settings to influence both PA and promote wider health benefits (Spotswood *et al.*, 2021).

In addition to PA, dietary behaviours also pose a challenge to health outcomes with a mean intake of fruit and vegetable consumption throughout the UK falling short of the recommended 5 portions per day across all ages and sexes (Public Health England, 2020). At the same time, childhood obesity rates, reported through the National Child Measurement Programme, have recently seen their sharpest annual increase since reporting began (NHS Digital, 2021). The Office for Health Improvement and Disparities (2022) has identified improved nutrition and PA as primary interventions to facilitate healthy weight status and schools have been described as an ‘ideal location for lifestyle modification interventions’ as they have dedicated time and facilities to support children to regularly engage in PA and nutrition education (Keats *et al.*, 2021). Nutrition interventions within school settings give children opportunities to develop their knowledge and selection of healthy foods at school, at home and in restaurants while supporting them to create a positive impression of food and nutrition (Kandiah and Jones, 2002). Moreover, the aim of nutritional interventions specifically targeting children is to create a positive impression of food and nutrition (Wagner *et al.*, 2005); while educating about the biological processes of food and its effects on the body to attempt to reduce any misconceptions (Slaughter and Ting, 2010). This early exposure to knowledge becomes key as children’s PA and dietary behaviours have been reported to track through to adolescence and adulthood (Mikkilä *et al.*, 2005; Telama, 2009).

Throughout childhood, parents/carers (subsequently referred to collectively as parents) have been identified as highly influential in children’s food choices and PA behaviours (Campbell and Crawford, 2001; Hennessy *et al.*, 2010). Parents are important social referents for children and adolescents (Bois *et al.*, 2005). Raudsepp (2006) identified parents can provide different types of social support in relation to PA for their children, ranging from instrumental and direct (e.g. finance, transportation), motivational (e.g. encouragement) and observational support (e.g. social modelling). While logistic support in the form of parents providing transport has been reported as one of the strongest social determinants (Hoefer *et al.*, 2001), the impact of parental modelling on children’s behaviour remains inconclusive (Sterdt *et al.*, 2014; Yao and Rhodes, 2015). Notwithstanding, systematic reviews have suggested that school-based interventions targeting PA, nutrition behaviours and obesity prevention can be more efficacious if a parental component is included (Kitzman-Ulrich *et al.*, 2010). Though, approaches to parental involvement in school-based interventions appear to be mixed and rely somewhat on information sent out to parents as opposed to direct involvement in activities (Tomayko *et al.*, 2021; Abderbwih *et al.*, 2022). Such messaging can become problematic when it is at odds with the health-related beliefs and practices that children encounter at home, leaving parents feeling judged and discriminated against (Spencer *et al.*, 2018). Therefore, the authors have also argued more needs to be done to understand parents’ health-related beliefs and practices and how these can have an impact on their children’s health. It has been further suggested that more research investigating parental involvement in health-related interventions is needed (Tomayko *et al.*, 2021; Abderbwih *et al.*, 2022).

### Current study

With the growth of PE outsourcing in schools in England, professional football (soccer) clubs, many of whom have independent football foundation charities (formerly referred to as football in the community), are a prominent provider (Parnell *et al.*, 2017). Football foundations are viewed favourably as they can connect with people in their communities who generally do not engage with conventional health interventions (Parnell *et al.*, 2013). However, research investigating the involvement of football foundations in schools is relatively limited (Parnell *et al.*, 2017). Furthermore, research exploring children’s and parents’ experiences of coach-led, health-promoting interventions is scarce. This is in line with arguments recently proposed by The British Academy (2022) highlighting views of children are seldom heard in relation to policies and interventions that affect them. Similarly, understanding of family experiences of PA interventions is lacking (Ross *et al.*, 2023), despite parents having a pivotal role in the development of children’s health behaviours (Karmali *et al.*, 2020). The problem with this lack of inclusion is that health-related interventions are designed and implemented without input from the groups they are supposed to help. Therefore, the current study aimed to qualitatively examine children’s and parents’ experiences of taking part in a PA and nutrition intervention delivered by an influential local football club foundation, looking specifically at their views of PA and healthy eating knowledge and practices.

## METHODS

### Approach

In order to understand children’s and parents’ experiences of the family football programme, the current study employed an interpretivist approach (Guba and Lincoln, 1994) which accounts for subjectivity while acknowledging the individual experiences of participants. Furthermore, a social constructionist epistemology was adopted as it focuses on interaction and social practices of participants where knowledge is socially constructed (Losantos et al., 2016). Moreover, from adopting a constructionist approach it is said that while the researcher acknowledges recurrence they also appreciate meaning as being central to the analysis process (Byrne, 2022). Aligned with the interpretivist approach, focus groups were adopted as they encourage rich insights into social exchanges between participants (Ryan et al., 2014).

### Background and the family football programme

The football foundation in the current study is an independent charity of a professional football club in the North East of England, UK. It delivers community-based health and education projects to promote healthier families. One of their programmes, family football is a free 6-week after-school programme for primary school children and their parents which runs for approximately 60 min once per week. While the programme is marketed and aimed at increasing PA and nutritional practices of children, the secondary aim is to initiate the transfer of this knowledge to the adult participants who accompany the children and engage in the activities alongside them. It is delivered by qualified sports coaches from the foundation which is registered as an independent charity in North East England. The foundation has established relationships with schools in the locality, and the programme is typically funded by schools that use either their primary school pupil premium or sports premium funding streams. The school’s role is to host the programme where they can use it to target children who struggle with engagement or behaviour with the aim to facilitate engagement between the school, children and parents within the school environment. Furthermore, schools can also choose to use the programme as a reward for children who have attained certain targets or are performing well. It should be noted that the structure and content of the programme remain the same irrespective of the criteria chosen by schools. Within the sessions, the groups contain parents and children from a particular key stage which can subsequently often include peers, friends and siblings.

Prior to commencing the programme, both children and parents/carers were assessed via a registration and health screening document. Staff also assessed the general mobility of participants and subsequently designed the practical sessions around these findings, for example for someone with reduced mobility, a football game could be substituted by walking football. Foundation coaches engage with parents and children in a classroom-based session to work on a series of tasks and quizzes designed around healthy eating, healthy lifestyles, well-being, teamwork and equality, diversity and inclusion (EDI). Knowledge exchange is facilitated via the delivery of an informational presentation and is supplemented by a workbook which is completed each week. Parents supported their children by assisting them with the workbook and offered motivational support during the taught session. After the classroom-based activities, children and their parents actively took part together in a practical element which included sporting activities and games (not limited to football). At the end of the year, families who have taken part in the programme are invited to attend a celebration event at the football club.

### Participants

A purposive sampling approach was utilized to recruit participants (*N* = 36 individuals) from a community-based family football programme delivered by a football club foundation (hereon referred to as the Foundation). Two schools who had completed the six-week programme during the spring term were randomly selected by Foundation staff to take part where parents and children from these sessions took part in focus groups. The final sample comprised 18 adults (one parent per child) (28–69 years of age SD = 12.89; 9 males, 9 females) and 18 children (6–9 years of age SD = 0.80; 12 boys, 6 girls). All parents self-reported they attended the programme with their child for at least 5 weeks of the 6-week course and were actively engaged in the programme.

### Materials

Prior to study commencement the research team met with the facilitators of the programme to gain an understanding of the delivery pattern and content. Subsequently, a semi-structured interview guide for parents and children was developed by the first and third authors to guide discussions about the programme.

### Procedure

The study received University ethical approval from the Faculty of Medical Sciences prior to study commencement and opt-in written consent was provided by parents for themselves and their children, with additional verbal assent obtained from all of the parents and children prior to focus groups taking place. Five focus groups were conducted in a relatively quiet common area prior to participants attending a family football celebration event. Parents and children took part in separate focus groups but were in plain sight of each other for the duration. Two researchers who were independent of the programme conducted the focus groups simultaneously.

### Analysis

The focus group data were analysed inductively using the six steps of reflexive thematic analysis (Braun *et al.*, 2016; Braun and Clarke, 2019) which in line with the interpretivist approach acknowledges the role of the researcher in the social construction of meaning. The first and second authors, who had prior experience of coding, familiarized themselves with the data transcripts and then extracted initial codes from the data where initial themes were then developed. Patterns of themes were then further developed to ensure meaningfulness and distinctiveness (Byrne, 2022) and once mutual agreement was reached between the first and second authors, themes were defined, named and a report produced.

## RESULTS

Participants reported the family football programme had a positive effect on children and adults who attended; both on their perceived PA levels and attitudes towards food and health. Analysis revealed two themes: ‘Healthy lifestyle facilitators and challenges’ and ‘Added value of programme participation’. Themes and subthemes are summarized in Table 1. Themes are then presented in detail to reflect the views of parents (P) *and* children (C).

**Table 1:** Summary of themes and subthemes

Themes	Subthemes
Healthy Lifestyle Facilitators and Challenges	Sound knowledge of PA and nutrition Variability in behaviour change—breakfast, fewer unhealthy foods, more PA, fussy eating, time constraints Reciprocal encouragement Already engaged in sports
Added Value of Programme Participation	Fun and enjoyment Quality family time—different to usual Social time with other families and coaches Wider knowledge beyond family PA and nutrition

### Theme 1: Healthy lifestyle facilitators and challenges

When asked to reflect on the programme, parents and children discussed the clear focus on healthy lifestyles. Children in particular were knowledgeable about health and nutrition, discussing the benefits of specific nutrients and why healthy habits are important. Interestingly, the facts reiterated by children related to the content covered in the programme. Parents also felt that they had learnt about the importance of balance and variety in dietary habits.

If you pull on weights it gives you muscle on your arms, and makes your bones stronger (C2; Group 2)

Interesting to see the portions you’re supposed to be eating (P3; Group 1)

In terms of how this knowledge translated into behaviour, responses from parents and children varied. Some children talked about eating more vegetables and less junk food, motivated by the programme and direction from their parents. Though a minority of children said that they had made no changes to their diets following the programme. This was reiterated by parents, with some suggesting that their children were engaging in healthier habits such as eating regular breakfast and drinking more water than they had previously, whereas some parents said that trying to initiate changes had been difficult due to barriers such as children being ‘fussy’ with food and the higher cost of healthier food.

I do eat crisps. I used to have three packs a day but now only one a day- my mam [mother] told me to (C2; Group 3)

He won’t eat veg, he just doesn’t want it- nuggets, pizza, crisps, nothing with sauce in it- he knows now he should be eating it. I’m going to try and change his diet a bit now because he has started boxing now, I think he will (P5; Group 1)

When discussing potential improvements to the programme, parents suggested that bringing food into the programme sessions for children to try could facilitate positive changes in dietary habits. Furthermore, parents believed that working with the football club provided children with valuable role models who could encourage positive behaviour change. One parent described how their child had been encouraged to eat breakfast as a result of the programme:

Since we’ve been going to the programme and it’s been incorporated with the football and he knows now footballers do that and they have cereal every morning, so he wants to (P4; Group 2)

Interestingly, parents talked mostly about the perceived benefits of the programme for their children. However, involvement in the programme had also encouraged lifestyle changes amongst some parents as the physical elements of the programme had highlighted limitations to their own physical health and fitness.

I realised when I was trying to run about with the bairn [their own child] I could do with getting a bit more fit (P1; Group 1)

I’ve identified I need to quit smoking, and when I done family football I couldn’t even do half a lap around the hall without being out of breath (P4; Group 1)

Parents and children talked about how they had enjoyed the physical activities included in the programme and had since sought opportunities to engage in more physical activities, such as football, park visits and swimming outside of the programme.

We’ve started going to the park more and playing football because he’s enjoyed the session and he’s wanting to go out with the family (P4; Group 2)

Although a lack of time was highlighted as a significant barrier to PA for parents, they felt that the programme provided an accessible route into regular exercise, which they were encouraged to engage in by their children. Reciprocally, children were enthused by seeing their parents take part in exercise, which was novel as parents pointed out that after school sports typically involve children only.

I find it fun being able to do something with my parents (C1; Group 1)

Usually we just drop him off at football and leave him but this time we actually got involved (P2; Group 2)

Discussions with children revealed that they already take up opportunities to participate in sports through school and community-based activities beyond the family football programme. This was reiterated by parents who talked about their children’s involvement in activities such as boxing and dancing. However, the costs associated with sports-based activities for children could be a barrier to attendance for some families.

I do urm some boxing lessons outside of school (C4; Group 1)

Some activities what they charge you to take part in is ridiculous (P4; Group 1)

### Theme 2: Added value of programme participation

Parents and children talked favourably about the social opportunities that the programme had provided. Family time was identified as a particularly valuable element as parents and children got to spend regular quality time together, which they typically did not get at home.

We get to spend time with our family and play football together (C2; Group 1)

We’ve bonded more through the programme… when you do something they’re looking forward to it, you talk about it and encourage them, “well done”, and you get closer, doing something with their parents (P5; Group 1)

Parents also enjoyed seeing their children interacting with other families and the coaches, and children valued this opportunity too. For some parents, they had recognized a level of confidence in their children that they had not seen elsewhere.

It helps you with other parents because you have time to have a laugh about and learn other stuff with other parents (C3; Group 3)

I knew my son’s competitive but I didn’t realise he would be like that with the grown ups, it didn’t phase him at all, even when the dads were coming towards him he would still tackle him (P3; Group 2)

In addition, there was an emphasis on the fun and enjoyment that the programme brought. Some parents had been apprehensive about attending the programme, but after being encouraged by their children, they found they had enjoyed it. Though the competitive nature of some parents attending the programme was viewed unfavourably.

Just a bit of running around with your parent and messing about. I didn’t think I’d enjoy it, but I did. I just thought I couldn’t be bothered, let them crack on with it, but he enjoyed it, it was a laugh, it went quickly, it was good (P5; Group 1)

Finally, parents and children commented on the wider knowledge gained through the programme (i.e. knowledge beyond nutrition and PA applicable to the family context). They had learnt about different types of football and disability sports, which they found interesting. However, it was suggested by parents and children that more time allocated to physical activities and less time spent on classroom-based activities would improve the programme.

If you were doing writing all the time and sitting down, that’s not doing enough exercise (P4; Group 3).

## DISCUSSION

The main aim of the present study was to understand parents’ and children’s perceptions of taking part in a family football programme. This study to our knowledge provides a first attempt to examine children’s and parents’ simultaneous experiences of participating in a PA and nutritional intervention, which has an indirect aim to target and influence future parental behaviours. Overall, the findings revealed two key themes of *healthy lifestyle facilitators and challenges* and *added value of programme participation*. The findings highlight the value that external providers can have in terms of engaging children and their parents. Notably, children were able to demonstrate knowledge of programme content in terms of healthy eating and both parents and children expressed how the programme enabled them to spend quality time with one another outside the challenges of everyday life. The findings therefore add some insight into how externally delivered programmes can produce positive outcomes while potentially relieving some of the pressure on schools to deliver effective health-related interventions within the curriculum.

Previous research has reported that both family and friend support can influence PA, and this dual approach to increasing PA can be effective as children get older (Thompson *et al.*, 2010). However, a key strength of the family football programme pertains to the inclusion of both parents and children within a focused PA and nutrition-based intervention delivered by Foundation coaches. While children were able to participate in the programme alongside their friends and peers this did not appear to be a key factor in their enjoyment. Indeed, a key finding from the current study pertained to both parents and children identifying that a significant benefit was being able to spend quality time together outside of their home environment. Typically, many children’s PA and nutrition interventions occur in schools whereby children take part independently of their parents with the support of teachers. This more traditional approach often removes parents from the process, with parents receiving information from their children who have taken part perhaps in the form of leaflets or letters from schools where useful information may not subsequently be acted upon (Spencer *et al.*, 2018; Abderbwih *et al.*, 2022). Notwithstanding, parents/guardians have been identified as one of five key stakeholders in the Creating Active Schools Framework (CAS; Daly-Smith *et al.*, 2020) with acknowledgment of the role they play in supporting children to engage in extracurricular activities. Indeed, in an Australian study, parental modelling and encouragement in activity have been identified as a key indicator of uptake of PA and also of sedentary behaviours in pre-school children (Dwyer *et al.*, 2008). Interestingly, in the current study, parents suggested they drew encouragement from their children to engage in the programme and were motivated to improve their own health, by engaging in behaviours such as trying to quit smoking, to allow them to keep pace with their children. This highlights the potential reciprocal advantages of parents and children taking part in health-related interventions together as opposed to a focus on one-way parent modelling. Furthermore, parents and children in the current study enjoyed spending time together engaged in a shared activity. With sports identified as an activity that can facilitate positive interaction between children and parents (Ginsberg, 2007), opportunities to engage in shared activities could confer additional benefits beyond physical health and nutrition.

Enjoyment was consistently discussed by children and parents throughout the focus groups. They enjoyed the activities offered by the programme as well as the social opportunities they were afforded. Defined as ‘a proactive behavioral and psychological process towards the eudaimonic or hedonic qualities of positive feelings’ (Kawabata & Mallett, 2022), enjoyment is well-established as a key motivating factor in children’s and adolescent’s sports and PA participation that can make a difference to whether activity involvement is sustained over time (Hagberg *et al.*, 2009; Gao *et al.*, 2013; Visek *et al.*, 2015). In their model of sport enjoyment, Scanlan and Lewthwaite (1986) relate enjoyment to (i) individual perceptions of personal ability; (ii) perceptions of competence drawn from social evaluation from others; (iii) physical sensations of movement and competition; and (iv) positive interactions with peers and adults. As well as offering opportunities for skill development, movement and competition, family football provided valuable opportunities for social interaction, which was a key source of enjoyment for children and parents in the current study. Moreover, it has been reported that parents are highly influential in younger children’s enjoyment of sports and can increase motivation and self-esteem through positive interactions and social support (McCarthy *et al.*, 2008). This was reflected in the current study as children gained a lot of enjoyment from taking part in activities alongside their parents. However, parental enjoyment is an area that warrants further investigation. Enjoyment in the current study appeared to be reciprocal with parents motivated to take part as they enjoyed joining in with their children. This opportunity to join in sports with their children was said to be a novel experience for parents as it is not something that is typically offered by out-of-school clubs.

Another unique element of the current programme stems from schools partnering with a football club foundation, which enhances the reach of programmes in the community. Associated with success and status, football can ‘reach and inspire its target audience in the way that no traditional educational actors can hope to achieve’ (Sanders *et al.*, 2014). In the current study, it is possible that the involvement of the football club acted as a motivator for parents due to their affiliation with the team. Moreover, allowing parents to work alongside their children meant they were experiencing the programme and retaining information for themselves. It has been proposed that shifting away from traditional learning and incorporating football-based, real-world examples into community football programmes, in the way that the current programme did, can be advantageous in making learning more accessible and relatable for wider audiences (Sanders *et al.*, 2014). Moreover, the inclusion of football-based activities in particular could be beneficial for schools to explore. In the current study, references were made to the enhancement of children’s confidence, which is consistent with previous research that has associated football participation with greater improvements in outcomes of psychological well-being, including confidence and self-esteem (Seabra *et al.*, 2013; Appleton, 2017).

While parents in the current study talked favourably about the programme, they recommended that the provision of food for children to try within sessions would enhance the programme. This idea is consistent with research into children’s taste preferences, which suggests that repeated opportunities to try unfamiliar and previously disliked foods can enhance children’s liking for those foods, which is an important factor in food intake (Cooke, 2007; Lakkakula *et al.*, 2010). However, funding might be a challenge for the implementation of food within programme sessions. While programmes that encompass an experiential element via food service providers to increase exposure and accessibility can be beneficial, funding and resources become paramount for implementation (Charlton *et al.*, 2021) and this may extend beyond the remit of primary school pupil and sport premiums and require larger investment.

While a strength of this study pertains to a real-world view of a PA and nutrition intervention aimed at children and their parents/carers being delivered by foundation coaches, it is to be noted that this approach could also bring some limitations. Most notably, some of the parents and children who attended the programme and participated in our study reported they were fans of the football club who delivered the programme and therefore, despite informing participants we were independent of the programme and focusing on programme-specific questions we cannot rule out that this may have led to some social desirability in responses. The current research was also held prior to the celebration event at the stadium which could have also led participants to view the programme more favourably.

Some of the children involved in the programme also took part in other sports, which brings into question the reach of the programme. Research has shown that barriers to PA and sports participation exist for various groups, including children with disabilities and those classed as overweight or obese (Sheilds *et al.*, 2012; Stankov *et al.*, 2012). In addition, while the programme used football as a conduit to promote PA and healthy eating, this could also act as a barrier to uptake for children and parents who do not like football or are hesitant due to its reputation for reflecting and perpetuating inequalities, misogyny, racism, homophobia and Islamophobia (Cashmore and Cleland, 2012; Kilvington *et al.*, 2022; Pope *et al.*, 2022). Future research should therefore consider the motivating factors behind programme participation for parents and their children and whether participation could be beneficial for typically excluded groups. Furthermore, while the current findings highlight the short-term gains from participation, it would be useful for future studies to determine longitudinal effects. Though, long-term investment in evaluation can be costly and is often beyond the limits of available funding for interventions (National Institute of Economic and Social Research, 2021). Additionally, evaluations of interventions involving children and families need careful planning to ensure measures are not too invasive and do not encroach on intervention activities (Ross *et al.*, 2023).

## CONCLUSION

Notwithstanding these limitations, the current findings highlight that a 6-week programme can have beneficial effects for children and parents who participate in terms of accessing a programme to enhance nutritional knowledge and PA participation within their own community and integrating it into family life. The programme is pitched as a school-based nutrition and PA programme aimed at children, and the findings indicate a positive experience for both children and their parents with some evident transfer of programme knowledge to adult participants. This demonstrates that programmes that involve well-known sporting organizations who provide a key role in the community as the hook to initiate attendance can provide a unique and useful platform. This can subsequently be used to engage parents specifically, who would not necessarily sign up for a programme of this nature. Results also highlight the need to ascertain the longevity of such knowledge and potential barriers and facilitators to further engage parents and their children in related programmes.

## Data Availability

Due to the nature of this research, participants of this study did not agree for their data to be shared publicly, so supporting data is not available.
